# Construction of a Conductive Polymer/AuNP/Cyanobacteria-Based Biophotovoltaic Cell Harnessing Solar Energy to Generate Electricity via Photosynthesis and Its Usage as a Photoelectrochemical Pesticide Biosensor: Atrazine as a Case Study

**DOI:** 10.1021/acsomega.3c10308

**Published:** 2024-03-27

**Authors:** Mustafa Buyukharman, Ibrahim Ender Mulazimoglu, Huseyin Bekir Yildiz

**Affiliations:** † Department of Physics, Faculty of Science, Istanbul University, TR-34134 Istanbul, Turkey; ‡ Department of Chemistry, Ahmet Kelesoglu Education Faculty, Necmettin Erbakan University, TR-42090 Konya, Turkey; § Department of Mechanical Engineering, Faculty of Engineering Architecture and Design, Bartin University, TR-74100 Bartin, Turkey; ∥ Photo-Electrochemical Systems and Materials Research Group, The Central Research Laboratory-Research and Application Center, Bartin University, TR-74100 Bartin, Turkey

## Abstract

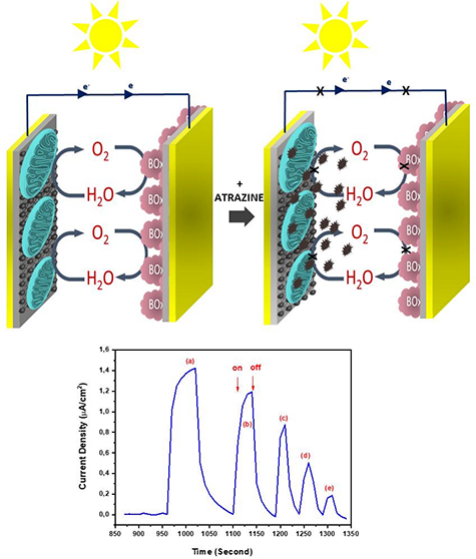

In this research, a cyanobacteria (*Leptolyngbia* sp.)-based biological photovoltaic cell (BPV) was designed. This clean energy-friendly BPV produced a photocurrent as a result of illuminating the photoanode and cathode electrodes immersed in the aqueous medium with solar energy. For this purpose, both electrodes were first coated with conductive polymers with aniline functional groups on the gold electrodes. In the cell, the photoanode was first coated with a gold-modified poly 4-(2,5-di(thiophen-2-yl)-1H-pyrrol-1-yl)benzamine polymer, P(SNS-Aniline). Thioaniline-functionalized gold nanoparticles were used to provide a cross-link formation with bis-aniline conductive bonds with the conductive polymer using electrochemical techniques. *Leptolyngbia* sp., one of the cyanobacteria that can convert light energy into chemical energy, was attached to this layered electrode surface. The cathode of the cell was attached to the gold electrode surface with P(SNS-Aniline). Then, the bilirubin oxidase (BOx) enzyme was immobilized on this film surface with glutaraldehyde activation. This cell, which can use light, thanks to cyanobacteria, oxidized and split water, and oxygen was obtained at the photoanode electrode. At the cathode electrode, the oxygen gas was reduced to water by the bioelectrocatalytic method. To obtain a high photocurrent from the BPV, necessary optimizations were made during the design of the system to increase electron transport and strengthen its transfer. While the photocurrent value obtained with the designed BPV in optimum conditions and in the pseudosteady state was 10 mA/m^2^, the maximum power value obtained was 46.5 mW/m^2^. In addition to storing the light energy of the system, studies have been carried out on this system as a pesticide biosensor. Atrazine biosensing via the BPV system was analytically characterized between 0.1 and 1.2 μM concentrations for atrazine, and a very low detection limit was found as 0.024 μM. In addition, response time and recovery studies related to pesticide biosensor properties of the BPV were also investigated.

## 1 Introduction

In light of the escalating global energy demand, the quest for novel, cost-effective energy sources has never been more pressing. Solar energy stands out as a paramount, boundless, and sustainable green energy source. Given the increasing energy requirements and growing environmental concerns, solar energy has emerged as a crucial alternative, especially when we consider the finite reserves of fossil fuels.^1^ Fossil fuel combustion, in addition to contributing to the greenhouse effect, exacerbates global warming, leading to undesirable climate changes.^2^ The imperative shift toward carbon-free energy production has become essential to mitigate the adverse impact of carbon dioxide (CO_2_) emissions from fossil fuel combustion.^3^ The astonishing and sustainable solar radiation reaching our planet in just 1 h possesses the potential to satisfy the entire annual energy consumption of the global population. One promising technology in this pursuit is biophotovoltaics (BPVs). Biophotovoltaics (BPVs), also referred to as photomicrobial fuel cells or microbial solar cells, represent an emerging technology aimed at converting solar energy into electrical energy through the utilization of photosynthetic microorganisms.^4,5^ For a normal microbial fuel cell, an organic substance is necessary in order to donate an electron to the system. However, BPVs use sunlight for the photolysis of water and provide electrons to the system via photosynthetic organisms.^6,7^ In contrast to traditional photovoltaic (PV) technology, BPV is considered more environmentally friendly, as the photosynthetic materials employed are nontoxic and renewable. Beyond generating electricity solely during daylight, BPV systems have the potential to produce electrical current in the absence of light by oxidizing intracellular metabolites.^8^ This stands in contrast to PV systems, which are inactive during the nighttime. Furthermore, BPV systems can function as energy-storage reservoirs, resembling rechargeable batteries with separable charging and discharging. This storage capability sets BPV apart from PV, which lacks the ability to store electricity.^9−11^ Consequently, BPV technology has garnered attention due to these notable advantages.

Many photosynthetic organisms are used for the biological photovoltaic cells.^12^ Among them, thylakoid membranes,^13^ purple bacteria,^14^ green algae,^15^ and cyanobacteria^6^ are the most preferred. The morphological diversity exhibited by cyanobacteria is indeed remarkable. Diverging from higher plants, cyanobacteria possess both photosynthetic and respiratory systems localized within thylakoid membranes in the cytoplasm. This unique arrangement facilitates the facile sharing of excess electrons generated during photosynthesis with the cytoplasm, albeit posing a potential risk of oxidative stress. Moreover, cyanobacteria have evolved a specialized mechanism to shield themselves from photodamage induced by high light intensities. This adaptive feature enables them to thrive across diverse environmental conditions. Importantly, the utilization of cyanobacterial cells in such systems obviates the need for intricate isolation processes. The intricate interplay between the photosynthetic and respiratory systems in cyanobacteria not only underscores their adaptability but also underscores the efficiency of their electron transfer processes. This nuanced physiological strategy further contributes to their resilience and survival under varying environmental challenges. Therefore, cyanobacteria are particularly favored for power generation in BPVs due to their high efficiency, direct energy production capabilities, and environmental compatibility.^16^

A multitude of cyanobacteria-based BPV studies have been conducted recently. Examples include *Geobacter sulfurreducens*,^17^*Synechococcus* sp. PPC 7942, *Anabaena variabilis*,^18^*Synechocystis* sp. PCC 6803,^19^ and *Shewanella oneidensis*(20) coated on graphite, carbon brush, Pt, Au, and indium tin oxide glassy electrodes.^21^ These studies generate a photocurrent by facilitating fast electron transfer. In studies conducted by our group, photocurrents were generated through fast electron transfer by connecting *Lyptolyngbia* sp.-type cyanobacteria on Os complex-modified polymers and conductive polymer/Au nanoparticle-type structures.^22^ Additionally, Howe and his research group successfully generated electricity in both light and dark environments without using a mediator in BPVs, achieved by creating cyanobacteria-based biofilms.^6^ Reports have underscored that the sustained presence of cyanobacteria in BPV cells over extended periods enhances the effectiveness of power generation facilities. They are widely used with artificial redox mediators in biological photovoltaic cells (BPVs) to transfer electrons from green cyanobacteria to the electrode in photosynthetic electricity generation. However, the use of nonenvironmentally friendly mediators to accelerate electron transfer in BPVs, together with these cyanobacteria, limits the use of these cells.^23^

At present, in order to get fast electron transfer and high photocurrent in solar cells, many biological or electronic transport materials such as cytochrome c, DNA, dye, metal oxides, calixarenes, and conducting polymers have been reported in the literature.^14,24−27^ Especially, in the realm of synthesizing and applying conducting polymers, a multitude of conjugated systems have been developed. Notably, 2,5-di(2-thienyl)pyrrole (SNS) stands out as a well-recognized member of this family. Introducing a pyrrole ring between two thiophene units enhances both the optical and the electronic properties of the resulting material. Specifically, SNS polymers (PSNS) exhibit lower oxidation potentials compared to their thiophene and pyrrole counterparts, facilitating easier oxidation and promoting improved stability. Furthermore, the unique structure of SNS allows for various substitutions around the N atom on the pyrrole ring, enabling precise adjustments to the electrochemical and optical characteristics of the conducting material.^28^ Due to these properties, P(SNS-Aniline) was used in this study.

Pesticides encompass a category of chemical substances used for fighting a range of factors, including plant diseases, detrimental insects, and weeds, which have the potential to diminish agricultural yields.^29^ According to their chemical structure, pesticides are classified as insecticides, fungicides, fungustatics, herbicides, acaricides, bactericides, aphicides, rodenticides, nematicides, molluscicides, algicides, avenicides, repellents, and attractants. Atrazine, a chlortriazine herbicide, is the most widely used herbicide to control weeds in agriculture due to its stable s-triazine structure, its effectiveness against a broader range of weeds, its ability to decrease susceptibility to soil erosion, and its cost-effective approach to inhibit the growth of targeted weeds^30^ by acting as an inhibitor of photosynthesis by specific binding to photosystem II.^31,32^ The effective use of these pesticides is very important, as insufficient usage often results in the survival of target pests, while excessive usage can damage crops and lead to the presence of pesticide residues in the natural environment. Of great significance is the fact that prolonged exposure to these contaminants such as atrazine, even at exceedingly low concentrations of just 1 ng/mL in drinking water, can lead to endocrine disruption and even cancer in human beings.^33−36^ Therefore, the determination of these chemicals in surface water and groundwater is becoming very important. Several conventional analytical techniques have been used for the detection of herbicides, such as gas chromatography,^36^ high-performance liquid chromatography,^37^ coupled mass spectrometry (HPLC-MS),^38^ and capillary zone electrophoresis.^39^ Although these techniques may provide efficient determination, they require high-cost and time-consuming protocols and extensive sample preparation procedures and require large amounts of samples and well-trained operators.

Today, biosensor design is promising in pesticide determination studies. Biosensors have gained attention in environmental monitoring for their ability to provide fast detection time, cheapness, use smaller sample volumes, and offer lower limits of detection for trace concentrations.^40,41^ Several highly selective, sensitive, rapid, and cost-effective amperometric, potentiometric, colorimetric, and other microbial biosensors, which consist of whole cells as bioreporters through coupling with physicochemical transducers to produce signals for the specific analyte(s),^42^ were constructed and used for direct determination of several pesticides from different samples.^43−45^ Based on the amperometric detection of photocurrent or molecular oxygen produced by photosynthesis by cyanobacteria or green algae, which is a direct or indirect way to measure a photoelectric current, microbial-based electrodes have started to be studied as photoelectrochemical and electrochemical biosensors for rapid and cost-effective analyses for pesticide detection in waters and very recently are of increasing interest. Although there are even few original amperometric pesticide sensor studies which use electrodes based on cyanobacteria or green algae from whole cell groups, i.e., photocurrent produced as a result of photosynthesis, either directly, i.e., photoelectrochemically,^46^ or indirectly, i.e., electrochemically (the amount of oxygen produced as a result of photosynthesis),^47^ a study mentioning an application study of BPVs that convert sunlight into electricity through photosynthesis as pesticide sensors is almost nonexistent in the relevant literature.

In this study, a BPV, which generates a high photocurrent through photosynthesis based on *Leptolyngbia* sp., a cyanobacteria, was fabricated. A gold electrode (GE) surface was first coated with poly 4-(2,5-di(thiophen-2-yl)-1H-pyrrol-1-yl)benzamine (P(SNS-Aniline)) and then thioaniline-functionalized gold nanoparticles, AuNPs, were cross-linked with conductive bis-aniline bonds to the P(SNS-Aniline)-coated GE as a result of electropolymerization. Then, cyanobacteria matured in a special culture medium were coated on the P(SNS-Aniline)/AuNP structure-modified GE. Finally, cyanobacteria were cross-linked to the AuNPs via bis(sulfoaxinimidyl)suberate (BS_3_) (photoanode). This structure generated a high-degree photocurrent because of the very fast transfer of electrons, which arises as a result of the oxidation of water in cyanobacteria via photosynthesis under visible light, from the thylakoid membrane to the electrode. To complete the BPV, another GE (used as the biocathode) is modified by cross-linking the bilirubin oxidase (BOx) enzyme with P(SNS-Aniline). When the system was operated under a constant potential by illuminating the visible region with light, water decomposed by oxidation through photosynthesis done by cyanobacteria, and photocurrent occurred by the transport of electrons released by the oxidation of water to the anode. While oxygen was released as a result of the oxidation of water in the photoanode of the BPV, the cathode side reduced this oxygen gas into water via a bioelectrocatalytic way. When pesticides were added to the BPV, the amount of photocurrent obtained in the BPV decreased due to the inhibition of photosynthesis in cyanobacteria by pesticides. When the concentration of atrazine in the medium was increased, the photocurrent produced in the BPV continued decreasing and was not observed after a certain period of time. Thus, it was possible to use this cyanobacteria-based BPV as a cheap, highly sensitive, and fast-responding photoelectrochemical amperometric pesticide biosensor. The application of the cyanobacteria-based BPV created in this study as a biosensor that can determine pesticides in water in a cheap, practical, fast, and accurate way will be the first time, and this highlights the novelty of the study.

## 2 Materials and Method

### 2.1 Materials

The 4-(2,5-di(thiophen-2-yl)-1H-pyrrol-1-yl)benzamine (SNS-Aniline) monomer and its electrochemical homopolymer were synthesized as in previous works.^48,49^ Thioaniline-functionalized AuNPs were prepared according to literature procedures.^50,51^ The concentration of BOx in a stock solution was determined by absorbance measurement at 600 nm using a ε of 4800 M^–1^ cm^–1^.^52^ The final specific activity was found to be 40 U per mg of protein. Bis(sulfosuccinimidyl)suberate was purchased from Pierce. The *Leptolyngbia* sp. type of cyanobacteria, which is among photosynthetic microorganisms, was purchased from Carolina. The cyanobacteria used in this study were reproduced according to the literature.^51,53^ The modified Leonian’s agar (MLA) complex, which was used in previous studies and is generally preferred for cyanobacteria, was used as the medium. Incubation was carried out at room temperature in a low-ion-intensity environment, and a white fluorescence lamp with a photon power of 40 μmol was adjusted to 12:12 light/dark. Cells were centrifuged at 20 °C for 10 min at 4000 rpm, washed with the electrolyte, and then centrifuged again under the same conditions. The obtained *Leptolyngbia* sp. cells were resuspended with the same electrolyte solution (1 g/mL) and immediately used for photoelectrochemical measurements. All other chemicals were purchased from Sigma-Aldrich and were used as supplied. Ultrapure water from a Nanopure (Barnstead) source was used throughout this work.

### 2.2 Method

#### 2.2.1 Fabrication of the Photoanode and Biocathode for the BPV

**Scheme 1:**
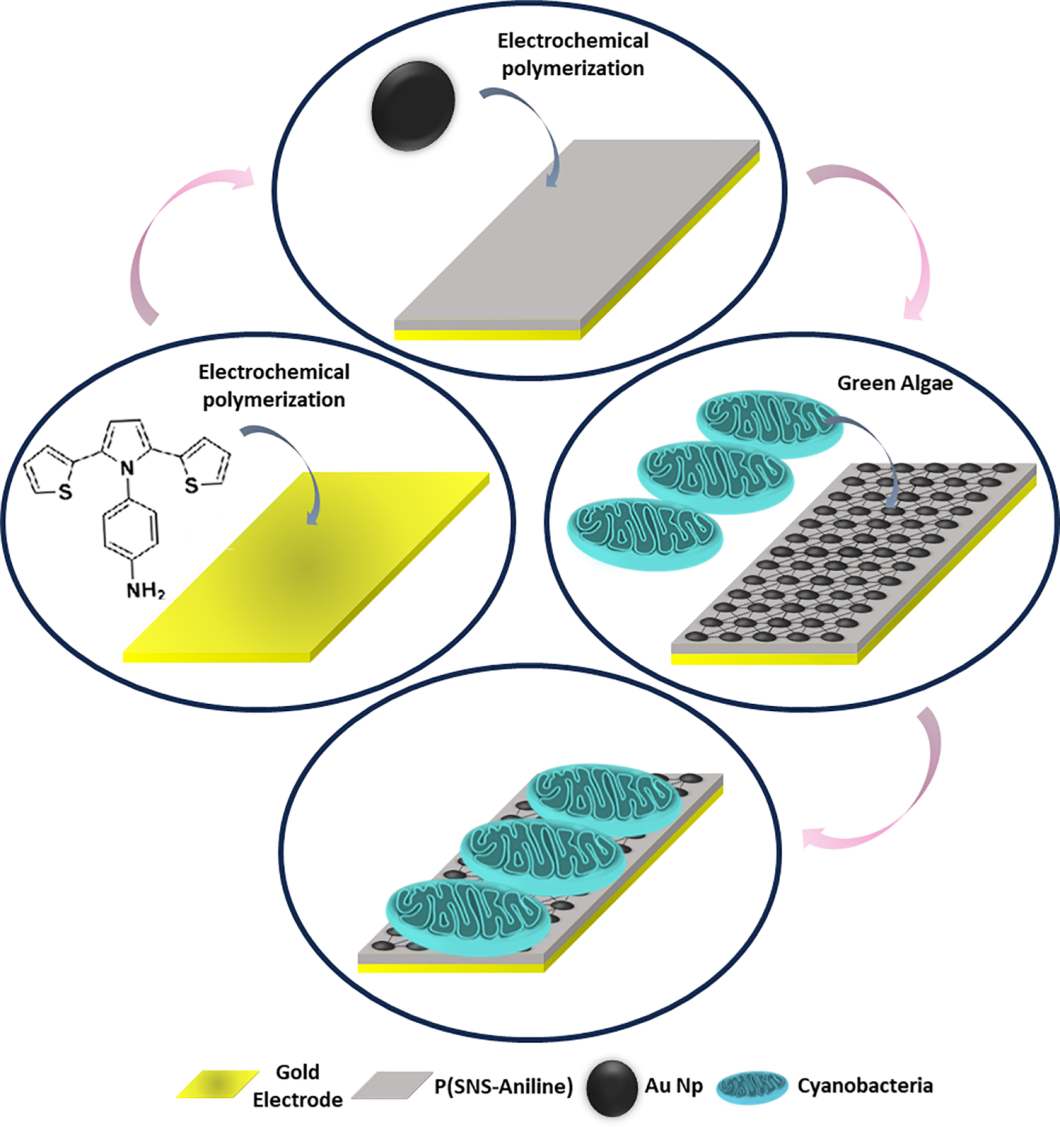
Schematic Representation for the Preparation of the Gold Electrode Coated with the P(SNS-Aniline)/AuNP/cyanobacteria Used as the Photoanode in the BPV

Before the modification, the gold electrodes, GEs, were mechanically cleaned with alumina slurry (0.5 μ) and washed with distilled water. The SNS-Aniline monomer was interacted with clean Au slides and polymerized electrochemically onto them by using a cyclic voltammeter in the −0.5 to 1.2 V potential range in the presence of a mixture of NaClO_4_ and LiClO_4_ in acetonitrile according to the literature.^48^ Thioaniline-functionalized AuNPs were electrochemically bonded with a P(SNS-Aniline)-modified GE using repetitive cyclic voltammetry scans in the potential range of −0.5 to 0.5 V in 0.1 M phosphate buffer, pH 7.4, according to the previous works.^50,51^ In the electropolymerization experiments, AuE as the working electrode, platinum wire as the counter electrode, and Ag/AgCl electrode as the reference electrode were used. After the electrochemical processes, the electrodes were washed with phosphate buffer and 100 μL of cyanobacteria (750 mg/mL) solution was added to the electrode surface. The cross-linking between the two materials occurred during the treatment of AuNPs and cyanobacteria with bis(sulfosuccinimidyl)suberate (BS_3_) solution (0.001 mg/mL) in 30 min and the construction of the photoanode was completed (Scheme 1).

**Scheme 2:**
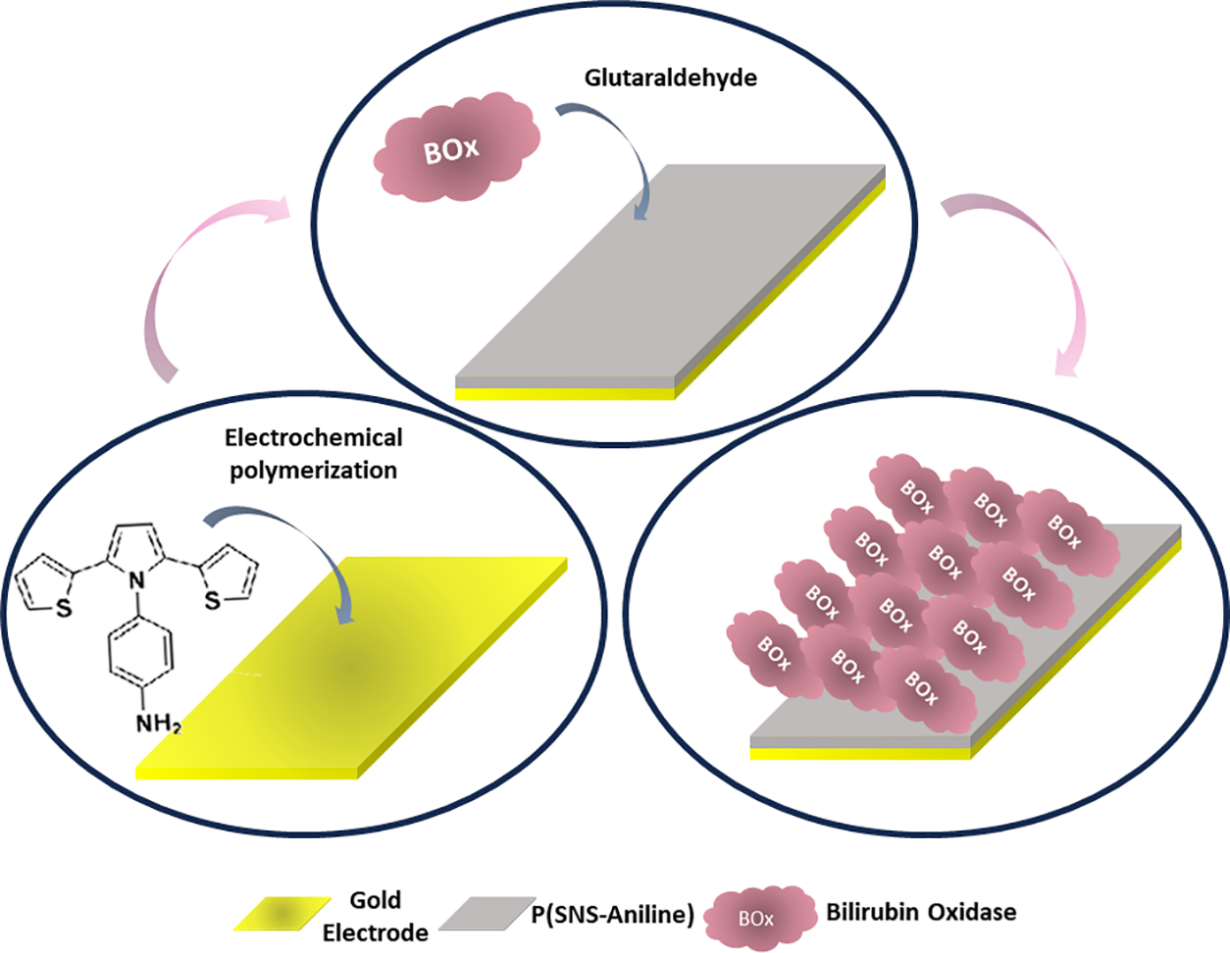
Schematic Representation of the Biocathode for the BPV The gold electrode modified with the P(SNS-Aniline)/BOx structure was designed.

For the biocathode preparation, in the same conditions as mentioned in the photoanode preparation, bare GEs were subjected to the same mechanical and electrochemical cleaning processes, and the SNS-Aniline monomer was electropolymerized on the GE as in the same conditions mentioned above, which is the preparation of the photoanode part. Finally, bilirubin oxidase (BOx) (1 mg/mL) and 10 μL of 1% glutaraldehyde were dropped onto the electrode surface (Scheme 2). Oxygen is one of the substrates of the BOx enzyme. The function of the BOx enzyme in this study is to reduce the oxygen gas produced via photosynthesis to water bioelectrochemically.^14,25,54^ Before being used, all electrodes were rinsed with distilled water to remove the unbound materials.

#### 2.2.2 Design of the BPV and Photocurrent Generation

The photocurrent experiments were performed via a solar simulator involving a special photoelectrochemical system. The instrumental system that was used during photoelectrochemical experiments consisted of an Oriel 300 W Xe lamp (Oriel Model 6258), a 2 nm-resolution monochromator (Model 74000), and a chopper (Model 76994). The translation of electrical data coming from the cell into digital data was performed by a phase-sensitive detector (Stanford Research Model SR 830 DSP). The current cut-release frequency was controlled by a pulse-delay generator manufactured by Stanford Research. For constant potential experiments within the scope of this study, a 3-electrode cell configuration, which contains the photoanode, platinum wire as the counter electrode, and the saturated Ag/AgCl electrode, and a potentiostat/galvanostat (EG&G Model 263) were used in addition to the electrodes mentioned above.

**Scheme 3:**
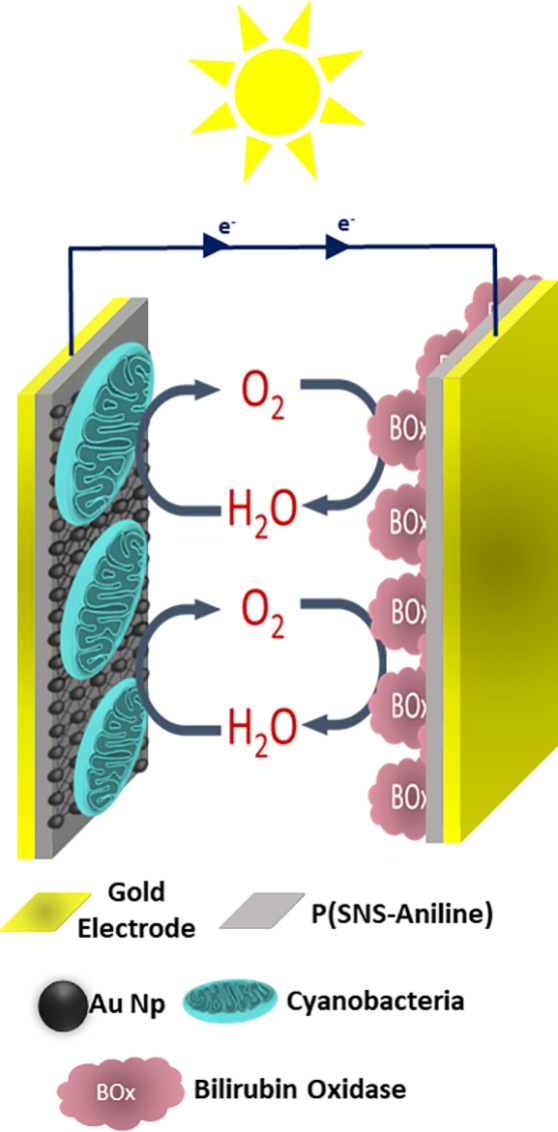
Schematic Representation of the Integration of the BPV

In photocurrent experiments, H-type cells were used. Electrodes (photoanode and biocathode) were put in the tubes having a distance of 50 mm between each other. In all experiments, 10 mM phosphate buffer at pH 7.4 (PBS) was used as the electrolyte. Both photoanode and biocathode electrodes are serially connected to the potentiostat device with different resistors (100 Ω to 10 kΩ). A multimeter, which was placed across the resistors, was used to measure the potential of the system. All photocurrent experiments were done via a potentiostat upon cyclic on–off illumination at a light intensity of 1400 W/m^2^ (1 sun unit) measured at the electrode surface. Before the measurement was conducted, electrolyte solutions were degassed with Ar gas for 15 min. When the system was operated under a constant potential by illuminating the visible region with light, water decomposed by oxidation through photosynthesis done by cyanobacteria and photocurrent occurred by the transport of electrons released by the oxidation of water to the anode. Conductivities of P(SNS-Aniline) and AuNPs, which act as a mediator, and conductive oligoaniline polymeric bridges between AuNPs and P(SNS-Aniline) accelerated the electron transfer from the cyanobacteria to the electrode in the system; therefore, the high-level photocurrent could be obtained under external visible light. While oxygen was released as a result of the oxidation of water in the photoanode of the BPV, the biocathode side reduced this oxygen gas into water via a bioelectrocatalytic way. In this established system, the potential difference on the multimeter indicated the electron current toward the P(SNS-Aniline)/BOx-modified gold electrode. The electron flow in the photoanode part is also proof of the oxygen gas formed. For the cathode side, the oxygen gas obtained was reduced to water again (Scheme 3). A constant applied potential (0 V) was used in chronoamperometry measurements vs Ag/AgCl in 0.1 M PBS, pH 7.4. All measurements were performed at a temperature of 20 ± 2 °C. All data reported here are averages based on three independent experimental repetitive data.

#### 2.2.3 Biosensing Characterization for the BPV

For the application of the cyanobacteria-based BPV as a pesticide sensor, calibration curves were obtained by plotting the photocurrent vs the substrate concentration and *y* = *mx* + *n* equations were obtained, where *y* is the sensor response in the current (nA) and *x* is the substrate concentration. The limit of detection (LOD) was calculated from the equations of LOD = 3*S*/*N* using the standard deviation of response (*s*) and the slope of the calibration curve. The atrazine detection from human tap water via the cyanobacteria-based BPV was performed by recovery tests after the addition of known amounts of atrazine to the tap water. No sample pretreatment was required for the analysis. The cyanobacteria-based BPV was incubated in 20 mL of buffer fortified with different concentrations of bisphenol A, arsenic, cadmium, copper, lead, and atrazine to see the synergistic effects of the chemicals in the mixture.

## 3 Results and Discussion

**Scheme 4:**
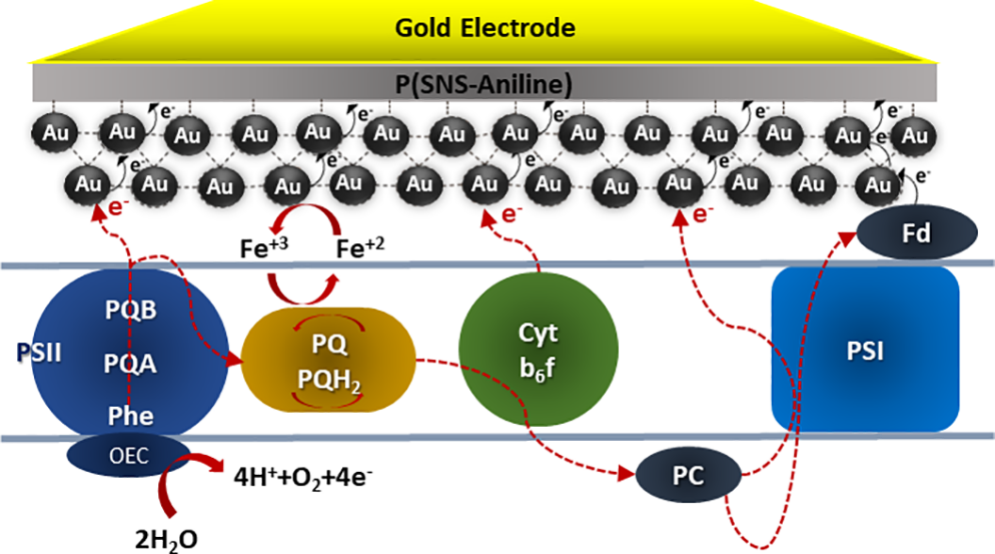
Schematic Representation of the Light-Dependent Electron Transfer Mechanism from the Reaction Center to the Electrode^22^

The proposed electron transfer (ET) facilitated by the conducting P(SNS-Aniline) and AuNPs is depicted in Scheme 4, illustrating the transfer of electrons from random cyanobacteria to the electrode. The schematic representation of extracellular electron transfer (EET) from photoheterotrophically grown cyanobacteria commences with the photo-oxidation of water, triggered by the excitation of Photosystem II (PSII) upon exposure to light photons. The electron transfer process progresses through a series of electron carrier quinone complexes (QA and QB), followed by subsequent electron transfer to Cyt b6f within the cytochrome, facilitated by a quinone cycle, as delineated in Scheme 3. Within the membrane, plastocyanin is postulated to undergo oxidation, serving as a conduit for electron transport to Photosystem I (PSI). Subsequent reactions induce a proton flow across the membrane, culminating in the generation of adenosine triphosphate (ATP). The mechanisms of electron transfer from the cell membranes of cyanobacteria to the surface of the electrode can be described in various ways, and further elucidation of these processes is crucial for a comprehensive understanding of the underlying electrochemical phenomena.^55^ The ET initiation occurs at Photosystem II (PSII), subsequently traversing the quinone pool (QA and QB) and PBQ and finally reaching the electrode. Mediating this process are P(SNS-Aniline) and AuNPs, serving as typical mediators that establish close interactions with cyanobacteria and molecular contact with the outer membrane. The redox-active sites within the polymer and AuNPs facilitate electron transfer to the electrode.^22^ An alternative pathway for electron transfer stems from a plausible oxidation–reduction mechanism. Photons absorbed by Photosystem I (PSI) or the P700 complex lead to the emission of electrons to an intermediary molecule known as iron(II) sulfide (FeS). Subsequently, these electrons are transferred to ferredoxin (FD), a crucial participant in the oxidation–reduction process of NADP^+^ to NADPH. The possibility of electron capture arises as P(SNS-Aniline)/AuNPs traverse the outer membrane pores, potentially reacting with charged molecules through transmembrane proteins located in the cytoplasmic membrane.^16,22^

**Figure 1:**
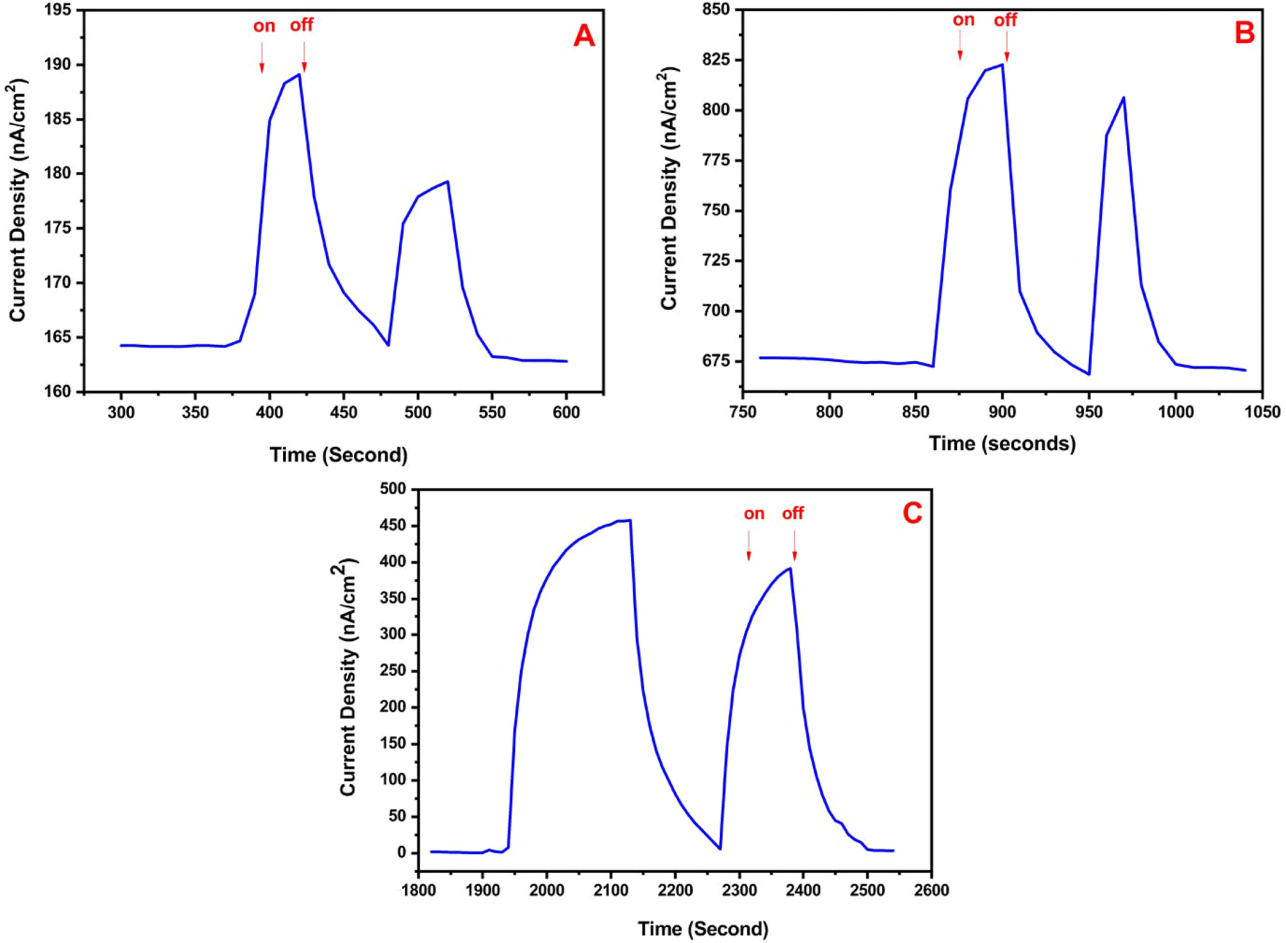
Control experiments of the photoanode in order to understand how the photocurrent was generated. (A) Chromatometry studies of the gold electrode coated with 500 mg/mL cyanobacteria in the presence of 0.410 μmol phenyl-*p*-benzoquinone. (B) Chronoamperometry studies of the gold electrode modified with the P(SNS-Aniline) (40 cycles)/cyanobacteria (500 mg/mL) structure. (C) Chronoamperometry studies of the gold electrode with the P(SNS-Aniline) (40 cycles)/AuNPs (40 cycles)/cyanobacteria (500 mg/mL) structure.

Several control experiments were performed in order to understand how the photocurrent is generated by the gold electrode modified with the P(SNS-Aniline)/AuNP/cyanobacteria structure. First of all, the cyanobacteria were immobilized on the gold electrode, and the cyanobacteria-coated gold electrode was immersed in the solution of phosphate buffer (pH = 7.4). When the light was sent to the system, it did not show any current. In the second photocurrent control experiment, a photocurrent (25 nA) was investigated when the cyanobacteria-coated gold electrode was immersed in phosphate buffer (the buffer solution with a pH of 7.4 has 0.410 μmol phenyl-*p*-benzoquinone solution) (Figure 1A). It has been implied that a mediator accelerating electron transfer is necessary for the electron transfer formed by the oxidation of water under photosynthesis. A constant potential of 0.0 V and a concentration of 500 mg/mL cyanobacteria were used for all control experiments.

In another control experiment, 500 mg/mL cyanobacteria were trapped in the gold electrode covered with the polymeric film after being coated with P(SNS-Aniline) as a result of 40 cycles using the gold electrode electropolymerization technique. When the gold electrode modified with the P(SNS-Aniline)/cyanobacteria system was dipped into the buffer solution and 1400 W/m^2^ visible region light was sent to this, the amount of photocurrent obtained under 0 V constant potential increased to 148 nA (Figure 1B). This phenomenon showed that the conductivity of the polymer film enhanced with the electron transfer with photosynthesis. In the last control experiment, after the conjugated polymer film was coated on the golden electrode with 40 electropolymerization cycles, 100 mM AuNP solution functionalized with aniline was attached to the polymer film with oligoaniline bonds using 40 electropolymerization cycles. After attaching 500 mg/mL cyanobacteria to this structure, the gold electrode coated with the P(SNS-Aniline)/AuNP/cyanobacteria system was dipped into the buffer solution and 1400 W/m^2^ visible region light was sent to the system. The photocurrent intensity was enhanced up to 481 nA (Figure 1C). This result shows that all cyanobacteria previously immobilized are not in electrical contact with the electrode before immobilization of AuNPs. Immobilization of AuNPs increases the amount of cyanobacteria electrically connected with the electrode. As a final control experiment, the gold electrode modified with the P(SNS-Aniline)/AuNP/cyanobacteria structure was immersed in pure ethanol, and visible light was sent to it. Photocurrent generation was not observed. This observation emphasized that the structure’s sensitivity is exclusive to water, and photocurrent generation hinges on the transfer of electrons, indicating a strong reliance on water as the electron source.

**Figure 2:**
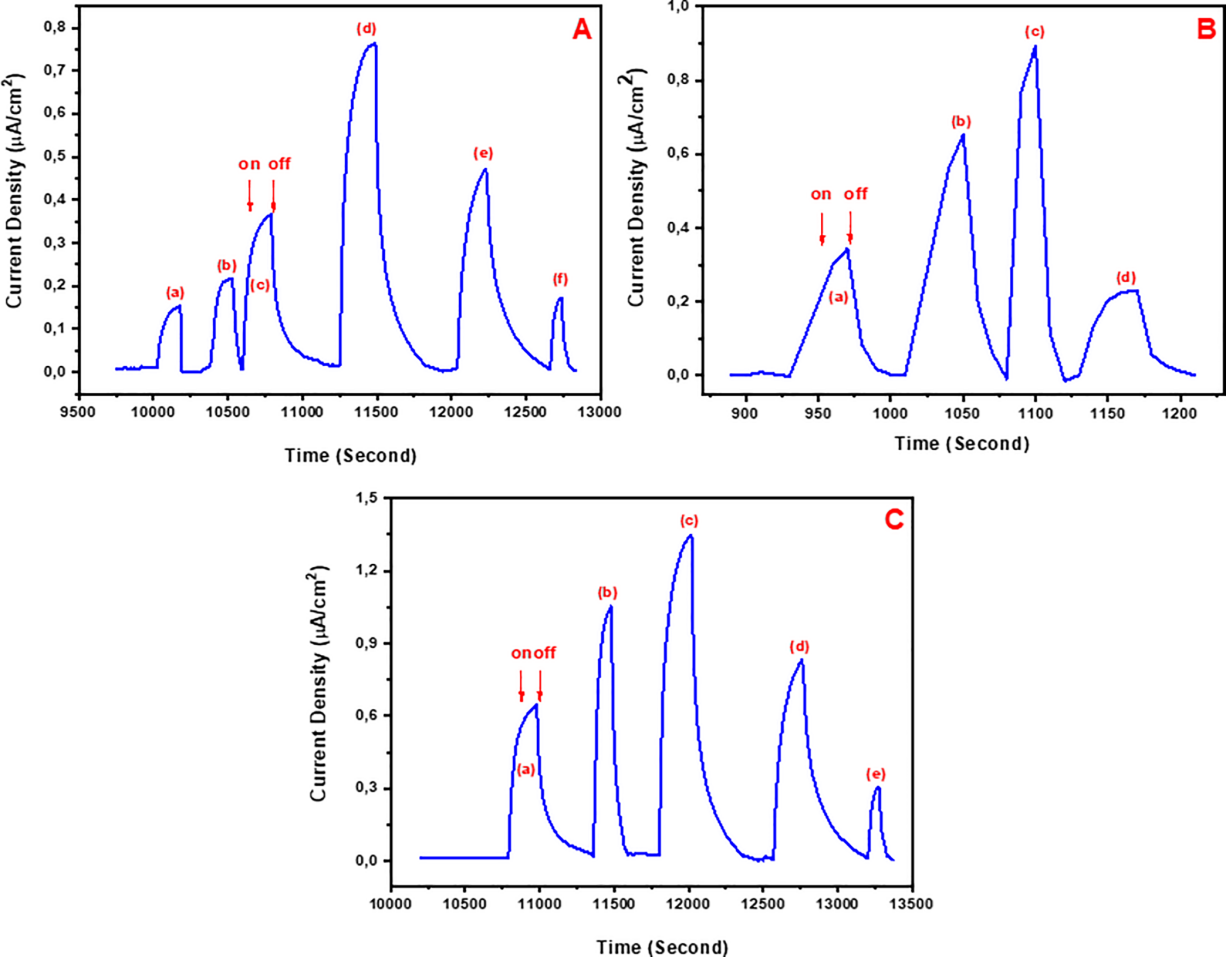
Chronoamperometry studies of the photoanode structure including (A) different cycles for AuNPs ((a) 20, (b) 30, (c) 40, (d) 50, (e) 60, (f) 70); (B) different concentrations of cyanobacteria ((a) 250 mg/mL, (b) 500 mg/mL, (c) 750 mg/mL, (d) 1000 mg/mL); and (C) different cycles for the conjugated polymer film ((a) 20, (b) 40, (c) 60, (d) 80, (e) 100).

One of the characterization studies of photoanode characterization is the number of cycle optimizations in cyclic voltammetry to connect AuNPs with the conjugated polymer film. The polymer prepared after 40 cycles was coated on the gold electrode. 100 mM aniline, which was functionalized with AuNP solution, was attached to the polymer film with the help of oligoaniline bonds using different numbers of cycles. Then, 500 mg/mL cyanobacteria were attached onto these structures. The photoanode electrode structure was dipped into the buffer solution under visible light, and various amounts of photocurrents were generated. When the number of cycles to bind AuNPs on the polymer film increased, the amount of photocurrent measured on the system also increased. This situation continued until 50 cycles, whereas the increase in the photocurrent stopped after 50 cycles, indicating the fastest electron transfer in the system. Despite the increase in the amount of AuNPs for the 60th and 70th cycles, the speed of electron transfer decreases since the rapidly increasing complexity of the conduction paths occurs in the system with the increase of the AuNP number. In this optimization experiment, 50 cycles were determined as the optimum number of cycles (Figure 2A). Another characterization study of the photoanode electrode is the optimization study for the amount of cyanobacteria. The P(SNS-Aniline) polymer prepared with 40 electropolymerization cycles was bounded with oligoaniline bonds to the conductive polymer film using 50 electropolymerization cycles of the aniline-functionalized AuNP solution (100 mM). Then, 250, 500, 750, and 1000 mg/mL cyanobacteria were immobilized on the P(SNS-Aniline)/AuNP modified GE. It has been observed that the photocurrent increases at best with a concentration of 750 mg/mL cyanobacteria. After this concentration value of cyanobacteria, the photocurrent decreased because of thickening of the biocomponent surface and prevention of the transfer of electrons to the electrode, which is formed by water oxidation as a result of photosynthesis (Figure 2B). Therefore, it is determined that 750 mg/mL is the optimum concentration for cyanobacteria to be used in the photoanode.

Another optimization study for the photoanode is to find the optimum thickness of the polymer film. In all experiments of the previous optimization study, the 40-cycled polymer film was used. In this study, the monomer was polymerized on the gold electrode in 20, 40, 60, 80, and 100 cycles by electropolymerization. On top of the polymer films obtained by these various cycle numbers, 100 mM aniline-functionalized AuNP solution was bounded with oligoaniline bonds using 50 electropolymerization cycles. Then, the immobilization of cyanobacteria (750 mg/mL) was experimented on the electrode. Thus, a photoanode for the system was obtained. It was produced by immersing this electrode in phosphate buffer (pH = 7.4) and by oxidizing water by photosynthesis under visible light. It was investigated that the highest value in the produced photocurrents was obtained for 60-cycled polymer films. The photocurrent value decreased when 80- and 100-cycled polymer electrodes were used. In cycles of more than 60, the electron transfer of the polymer film was not easy due to film thickness so that less amount of electrons reached the electrode (Figure 2C). For that reason, this cycle number for the polymer film was found as the optimum number of cycles.

**Figure 3:**
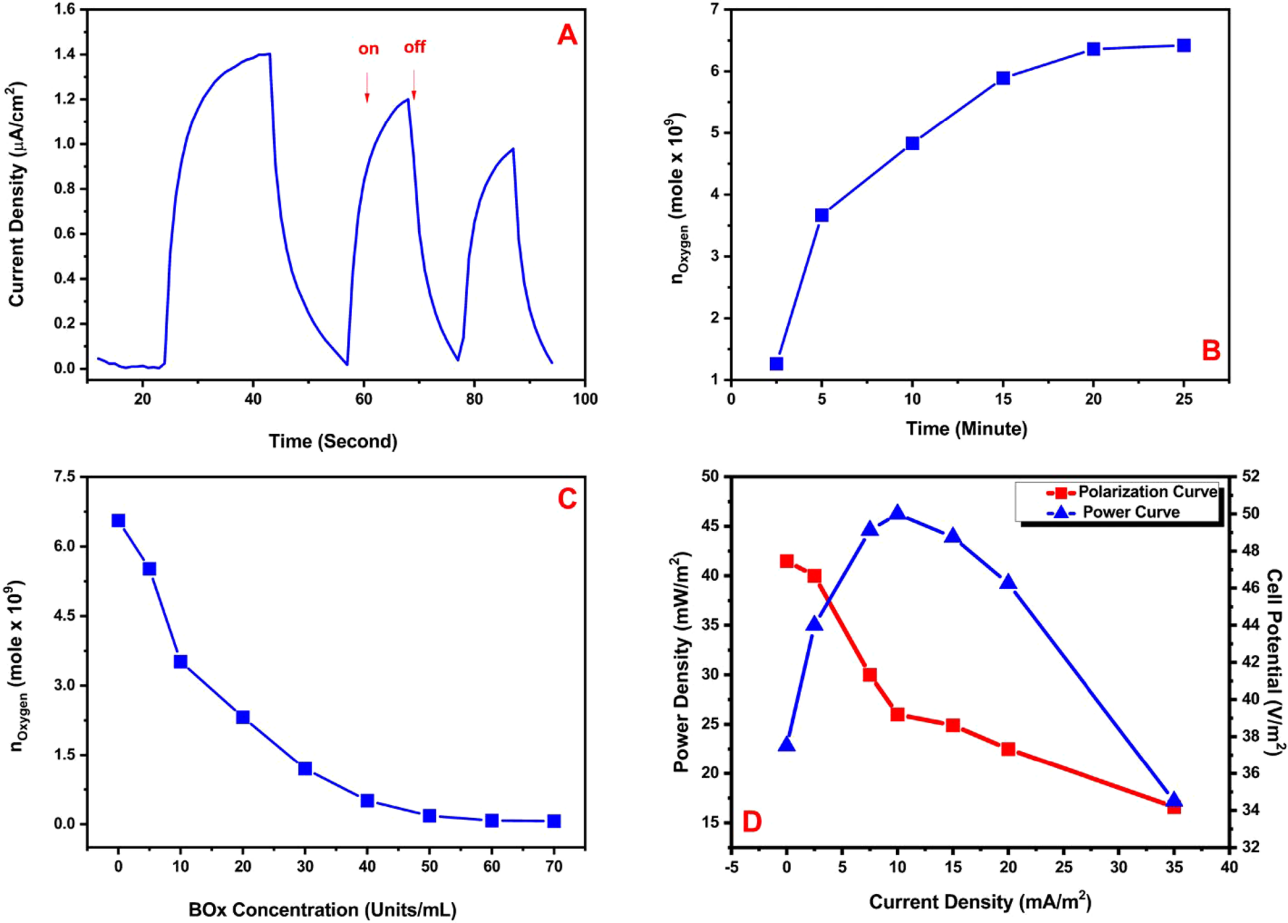
(A) Chromatometry experiments in order to show the interference effect of oxygen concentration during the photocurrent generation. (B) Anode electrode optimization according to oxygen concentration vs time. (C) Cathode electrode optimization in terms of BOx concentration vs oxygen concentration. (D) Polarization and power output curves.

After the optimum conditions were determined, another characterization study of the modified photoanode structure was performed on–off study. The P(SNS-Aniline)/AuNP/cyanobacteria-modified GE was illuminated with a fixed light source of 1400 W/m^2^ for some time, and then, the illumination was terminated. A decrease in the amount of photocurrent was obtained after the process was repeated each time (Figure 3A). The reason for this decrease in photocurrent is explained by the oxygen gas penetration by the oxidation of water. A Clark-type electrode system was performed in order to have an idea about how much amount of oxygen is generated in water as the illumination of the photoanode occurred for a certain period of time. Figure 3B shows obtained currents by illuminating the visible region under the BPV of 1400 W/m^2^ power for 2.5, 5, 10, 15, 20, and 25 min. After the system was illuminated for 25 min, the amount of oxygen generation by water oxidation as a result of photosynthesis by the gold electrode modified with the P(SNS-Aniline)/AuNP/cyanobacteria structure was calculated as 6.56 × 10^–9^ mol/cm^2^. The performance of the biocathode in the BPV was obtained by assaying the amount of oxygen in the system. The increase for the reduction of oxygen concentration is evaluated with the increase in BOx concentration. This indicates that the performance of the system depends on the biocatalytic functional property of BOx. The oxygen concentration showed a decrease little by little, and the equilibrium of the system was gained with 60 U/mL of BOx, indicating the reduction of max. oxygen gas on the cathode electrode (Figure 3C). The measurements for the anode can be summarized in conditions as the cycle number for P(SNS-Aniline):60, the cycle number for AuNPs:50, and the concentration of cyanobacteria: 750 mg/mL. In the case of the cathode electrode, the cycle number for the P(SNS-Aniline) film is 60. It is possible to say that the amount of oxygen in the BPV is directly reduced to a water molecule on the cathode surface. This also increases the energy output in the BPV system, and it also increases the lifetime of the cyanobacteria by excluding higher oxygen saturation during the cyanobacteria mechanism-related oxygen stress dependency conditions. The polarization curves were drawn to get data-related power generation of the BPV system (the black line in Figure 3D). The light conditions were chosen between 16.5 and 46.6 mW/m^2^. The maximum power output (46.6 mW/m^2^ with a current density of 10 mA/m^2^) was obtained. For the system to control including the growth media, when the cyanobacteria were not used, the results showed that there was no considerable current response during illumination. Just as it was expected, the system power increased by the light is exactly related with the change in the oxygen concentration in the BPV. The oxygen concentration in the reaction chamber is increased as predicted due to the photosynthesis done by the cyanobacteria (seen in the increase, as shown in Figure 3B). The cathode part helps reduce the oxygen amount in the reaction with the catalytic effect of adsorbed BOx (see the curve in Figure 3C). The power generation is related with the anodic interactions in the system. However, the system stabilization depends on the oxygen concentration equilibrium for longer periods in between both electrodes.^16^

**Figure 4:**
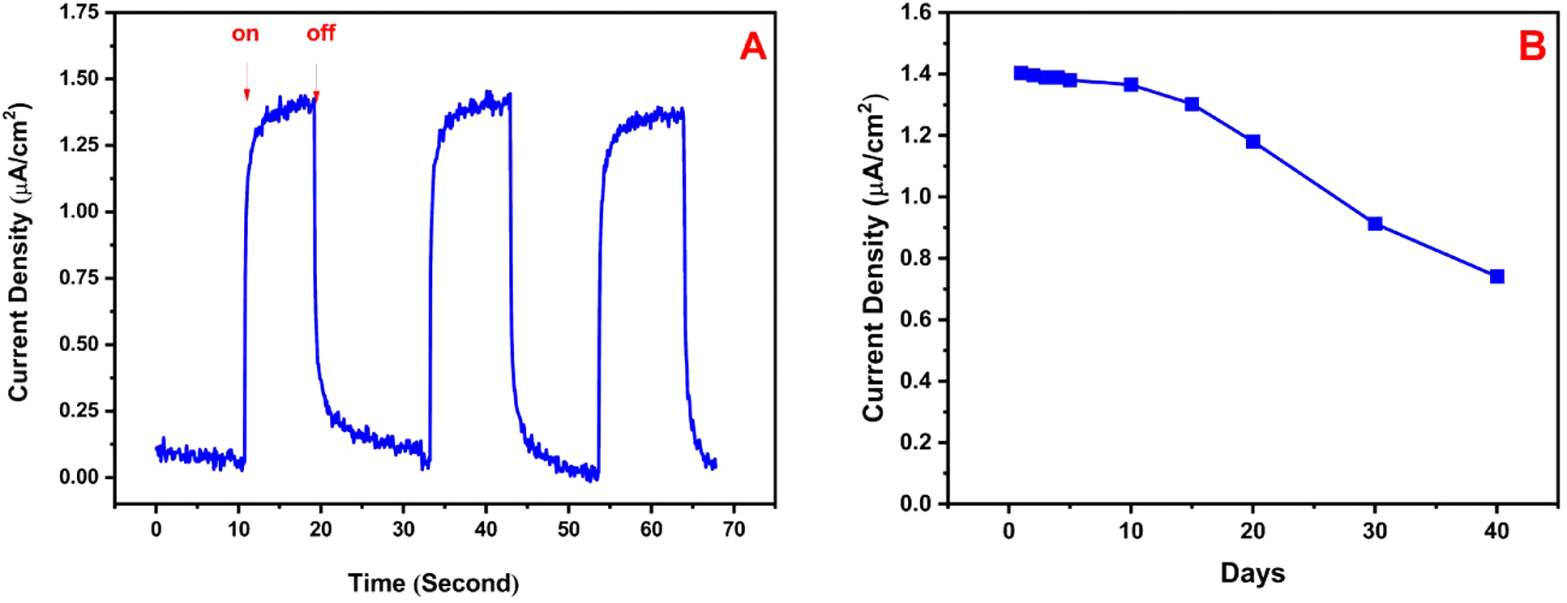
(A) Working and (B) storage stability studies of the cyanobacteria-based BPV.

The working stability of the BPV was performed with the help of chronoamperometry photocurrent measurements for a long period of time. Results showed that a high photocurrent activity of the BPV occurred up to 180 min, without any progressive decrease of photocurrent later on. Figure 4A shows 70 min chronoamperometric photocurrent measurements. This proves that it has very good operational stability and high intraelectrode repeatability. The storage stability of the BPV was also evaluated during 40 days. Chronoamperometry tests helped, and the photocurrent signals were recorded in time intervals (Figure 4B). No detectable photocurrent generation decrease occurred up to the 10th day. This can be expressed as the percentage of the generated photocurrent. A 92% of initial activity of cyanobacteria retained within 15 days (84% within 20 days, 65% within 30 days, and 53% within 40 days). It can be said that the cyanobacteria-based BPV has a very good storage stability.

**Figure 5:**
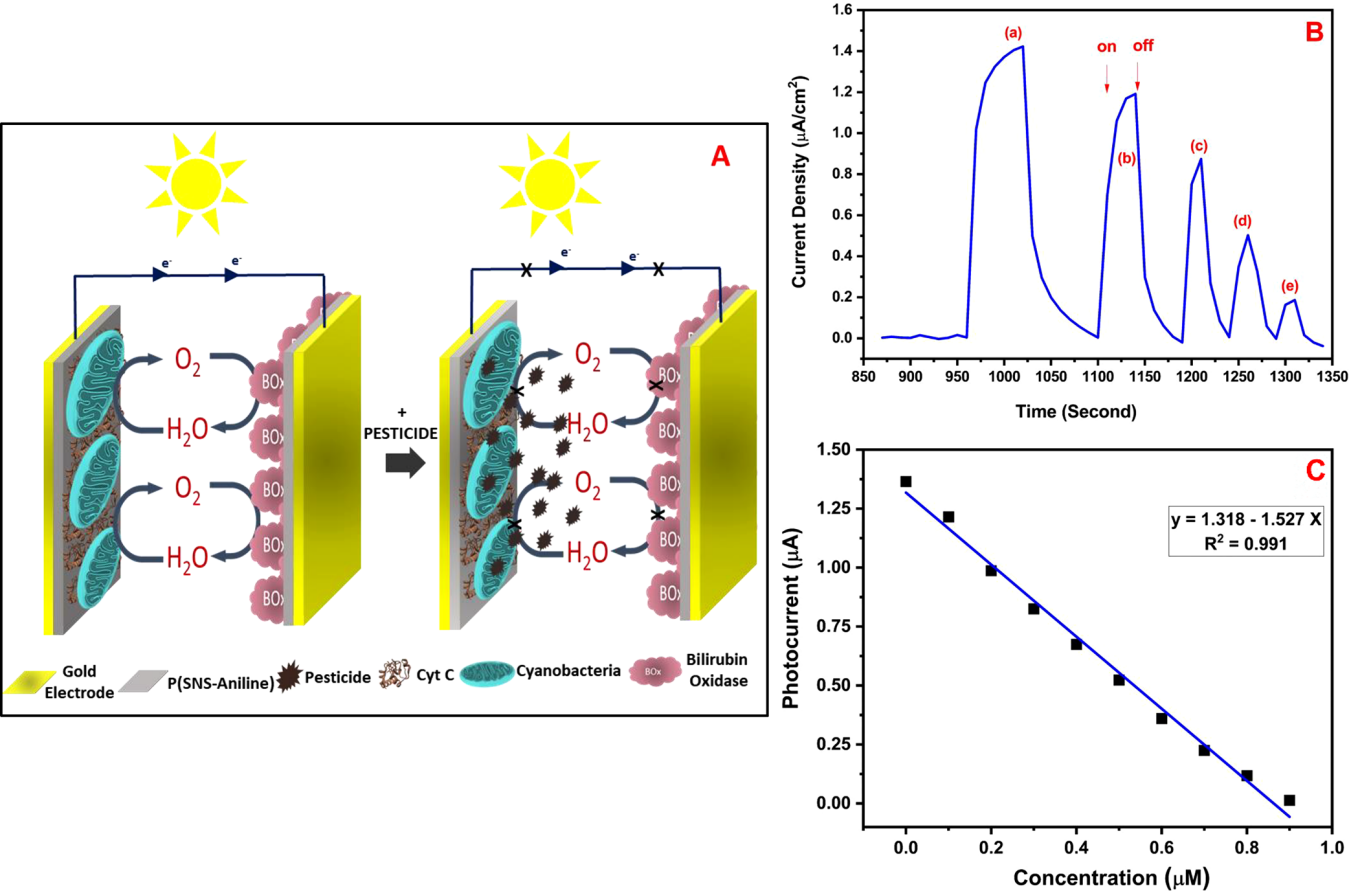
(A) Schematic representation of the biosensing approach of the BPV for pesticides in water. (B) On–off display of photocurrents produced by the oxidation of water as a result of photosynthesis through (pH = 7.4), under illumination for atrazine, with (a) 0 μM, (b) 0.2 μM, (c) 0.4 μM, (d) 0.6 μM, and (e) 0.8 μM concentrations. (C) Calibration graph of the biosensing approach of the BPV for atrazine.

When chemicals belonging to the pesticide class named atrazine and diuron were added to water, the photocurrent obtained exhibited a decrease due to the pesticides’ inhibition of photosynthesis in cyanobacteria. These pesticides bind to QA or QB. These inhibitors are bound to the QB complex with two possible electron carriers. Then, electron transfer is entirely stopped. When they bind with the QA complex, the transfer of electrons gradually decreases and is not completely stopped^32,43,44^ (Figure 5A,B). Figure 5 shows a comparison of the photocurrent values obtained using pesticides with and without inhibitors. The measurement conditions for the photoanode are summarized as follows: cycle numbers for polymer:60; cycle numbers for AuNPs:50; the concentration of cyanobacteria: 750 mg/mL. For the cathode electrode, the cycle number of P(SNS-Aniline) is 60 with 60 units/mL BOx with atrazine as obtained in 0.1 M PBS (pH 7.4) with different atrazine concentrations from 0.1 to 1.2 μM (with an illumination of 1400 W/m^2^). The cyclic on–off illuminations occurred with applied potential. It was evidently seen from figures that there was a photocurrent decrease with the effect of pesticides. When the concentration of pesticides increased, the photocurrent of the BPV decreased but was not completely shot off and some activity losses occurred for all chronoamperometry measurements. Therefore, it was possible to use the cyanobacteria-based biological photovoltaic cell, which produces electricity continuously, as a sensitive pesticide biosensor for atrazine (Figure 5B).

Under optimized conditions, atrazine and diuron biosensing was analytically characterized between 0.1 and 1.0 μM concentrations belonging to atrazine for the BPV system to be used. The LOD value was obtained for atrazine as 0.014 μM ± 0.0011 using the S/N ratio (Figure 5C). The corresponding LOD value of the proposed cyanobacteria-based BPV to be used as a biosensor for atrazine is too much lower than that of previously reported studies in the literature (Table 1). Furthermore, this cyanobacteria-based biophotovoltaic (BPV) system has exhibited significantly better results in terms of storage stability and response time compared to the studies mentioned in the literature in Table 1 for use as a biosensor.

**Table 1:** Comparison of the Performances of the Cyanobacteria-Based BPV to be Used as a Biosensor

electrode	detection limit for atrazine	storage stability	response time	ref
silicon septum/*Chlamydomonasreinhardtii*	95 nM (21 μg/L)	3 weeks	15 min	(56)
carbon black-screen-printed electrode/*Chlamydomonasreinhardtii*	1 nM (0.22 μg/L)	3 weeks (retaining 70% activity)	15 min	(47)
carbon felt electrode/aliginate/*p*-benzoquinone *Anabaena variabilis*	0.064 μM	n.d.	20 min	(46)
screen-printed electrode *Chlorella vulgaris*	200 mg/L	4 weeks 4 °C (retaining 80% activity)	4 min	(57)
*Leptolyngbia* sp. based BPV	0.014 μM = 3.08 μg/L	40 days (retaining 53% activity)	10 min	this work

The cyanobacteria-based BPV was incubated in 20 mL of buffer fortified with standard solutions of 10 ppb bisphenol A, 100 ppb arsenic, 5 ppb cadmium, 20 ppb copper, 10 ppb lead, and 0.4 μM atrazine. In addition to these chemicals, it was used to increase the synergistic effects of the chemicals in the mixture. The results were evaluated, and it was concluded that these mixed chemical materials did not affect the atrazine analyses in various concentration tests.

The recovery of the cyanobacteria-based BPV for atrazine was evaluated by adding 0.5 μM atrazine to the tap water samples already containing 2.5 μM. Table 2 shows the recovery result, and the biosensor exhibited perfect recovery even for a low concentration of the atrazine sample.

**Table 2:** Recovery Result of the Cyanobacteria-Based BPV to be Used for the Analysis of Atrazine

tap water (μM)	added material (μM)	detected material (μM)	recovery (%)	percentage (%)
2.5	0.5	3.01 ± 0.011	100.4	2.2

## 4 Conclusions

In this study, a cyanobacteria-based biological photovoltaic cell (BPV) was designed in order to generate photocurrent by illumination of the electrode system in an aqueous solution medium. It was also used as a biosensor for the detection of pesticides. For this aim, one of the electrodes was coated with a conjugated polymer (P(SNS-Aniline)) via the electropolymerization method. Then, it was covalently linked to the first AuNP via bis-aniline functional units, and a cross-linked structure between layers was formed. The *Leptolyngbia* sp. cyanobacteria were bounded to this newly constructed electrode surface with the help of BS3. This electrode, which is formed by stacking different layers on a gold electrode, acts as a photoanode in the cell. In the cathode construction, another conductive polymer, P(SNS-Aniline), was electrochemically coated on a second new gold electrode and then this new polymer was first activated with glutaraldehyde and then immobilized with the bilirubin oxidase enzyme via cross-linking. BPV was successfully worked under illumination, and water was oxidized and split photosynthetically. Oxygen gas formation was obtained on the photoanode, and at the same time, the cathode electrode reduced the oxygen into water via the bioelectrocatalytic method. In BPV, the role of AuNPs was very important during the binding of cyanobacteria to the electrode surface because of the fast electron transfer released as a result of photosynthesis. According to measurements, the optimum conditions were found in order to get a high photocurrent from the photoanode part of the BPV. The concentration of BOx for the biocathode electrode modified with P(SNS-Aniline) (60 cycles) was also optimized. The BPV was designed with a photoanode and a biocathode in the medium of water. The maximum power of the BPV was generated at a stable state with a current density of 10 mA/m2. At the working stability experiments of the BPV, a high photocurrent activity of the BPV occurred up to 80 min, without any progressive decrease of photocurrent later on and it was observed that the BPV has a very good operational stability and a high intraelectrode reproducibility. Storage stability experiments were also done in a volume of 10 mL of PBS (pH = 7.4) at the same conditions as other experiments during 40 days. The BPV has a very good storage stability because it could generate not only 92% of their initial photocurrent value within 15 days but also 53% within 40 days.

When chemicals belonging to the pesticide class named atrazine were added to the water, a decline in the amount of photocurrent generated in the photovoltaic cell was experimented due to the pesticides’ inhibition of photosynthesis in cyanobacteria. Atrazine and diuron biosensing via the BPV system was analytically characterized between 0.1 to 1.2 μM concentrations for atrazine, and a very low detection limit was found as 0.014 μM for atrazine. This BPV, which can be used as a pesticide biosensor, has successfully passed the real sample test application by showing very good sensor properties. Many advantages were highlighted for the proposed cyanobacteria-based BPV to be used for the biosensing of pesticides. It showed reproducibility, high sensitivity, and stability when it was compared with its biosensor analogues in literature. In the future, more advanced studies related to this study will be performed in order to analyze pesticide samples in domestic and industrial waters. This environmental application will be an easy, cheap, practical, fast, and sensitive measurement for pesticide analysis.

## Supporting Information Available

Detailed information about the synthesis and characterization of the (SNS-Aniline) monomer, homopolymerization of SNS-Aniline, and the synthesis and characterization of AuNPs. (PDF)

## Dedication

This article is dedicated to Mustafa Kemal Ataturk, who is the leader of the Turkish Nation and the founder of the Republic of Turkey.

## References

[ref1] Sekar N.; Ramasamy R. P. Recent advances in photosynthetic energy conversion. J. Photochem. Photobiol. C 2015, 22, 19–33. 10.1016/j.jphotochemrev.2014.09.004.

[ref2] Kruse O.; Rupprecht J.; Mussgnug J. H.; Dismukes G. C.; Hankamer B. Photosynthesis: a blueprint for solar energy capture and biohydrogen production Technologies. Photochem. Photobiol. Sci. 2005, 4, 957–970. 10.1039/b506923h. 16307108

[ref3] Lewis N. S.; Nocera D. G. Powering the planet: Chemical challenges in solar energy utilization. Proc. Natl. Acad. Sci. U.S.A. 2006, 103, 15729–15735. 10.1073/pnas.0603395103. 17043226 PMC1635072

[ref4] Howe C. J.; Bombelli P. Electricity production by photosynthetic microorganisms. Joule 2020, 4, 2065–2069. 10.1016/j.joule.2020.09.003.

[ref5] Wey L. T.; Bombelli P.; Chen X. L.; Lawrence J. M.; Rabideau C. M.; Rowden S. J. L.; Zhang J. Z.; Howe C. J. The development of biophotovoltaic systems for power generation and biological analysis. ChemElectroChem 2019, 6, 5375–5386. 10.1002/celc.201900997. 31867153 PMC6899825

[ref6] McCormick A. J.; Bombelli P.; Scott A. M.; Philips A. J.; Smith A. G.; Fisher A. C.; Howe C. J. Photosynthetic biofilms in pure culture harness solar energy in a mediatorless bio-photovoltaic cell (BPV) system. Energy Environ. Sci. 2011, 4, 4699–4709. 10.1039/c1ee01965a.

[ref7] Zhang Y.; Noori J. S.; Angelidaki I. Simultaneous organic carbon, nutrients removal and energy production in a photomicrobial fuel cell (PFC). Energy Environ. Sci. 2011, 4, 4340–4346. 10.1039/c1ee02089g.

[ref8] Tanaka K.; Tamamushi R.; Ogawa T. Bioelectrochemical fuel-cells operated by the cyanobacterium, Anabaena variabilis. J. Chem. Technol. Biotechnol. 1985, 35, 191–197. 10.1002/jctb.280350304.

[ref9] Liu L.; Choi S. PEDOT:PSS/MnO_2_/CNT ternary nanocomposite anodes for supercapacitive energy storage in cyanobacterial biophotovoltaics. ACS Appl. Energy Mater. 2020, 3, 10224–10233. 10.1021/acsaem.0c02054.

[ref10] Pankratova G.; Bollella P.; Pankratov D.; Gorton L. Supercapacitive biofuel cells. Curr. Opin. Biotechnol. 2022, 73, 179–187. 10.1016/j.copbio.2021.08.008. 34481244

[ref11] Ter Heijne A.; Pereira M. A.; Pereira J.; Sleutels T. Electron storage in electroactive biofilms. Trends Biotechnol. 2021, 39, 34–42. 10.1016/j.tibtech.2020.06.006. 32646618

[ref12] McCormick A. J.; Bombelli P.; Bradley R. W.; Thorne R.; Wenzel T.; Howe C. J. Biophotovoltaics: oxygenic photosynthetic organisms in the world of bioelectrochemical systems. Energy Environ. Sci. 2015, 8, 1092–1109. 10.1039/C4EE03875D.

[ref13] Cho Y. K.; Donohue T. J.; Tejedor I.; Anderson M. A.; McMahon K. D.; Noguera D. R. Development of a solar-powered microbial fuel cell. J. Appl. Microbiol. 2008, 104, 640–650. 10.1111/j.1365-2672.2007.03580.x. 17927750

[ref14] Cevik E.; Carbas B. B.; Senel M.; Yildiz H. B. Construction of conducting polymer/cytochrome C/thylakoid membrane based photo-bioelectrochemical fuel cells generating high photocurrent via photosynthesis. Biosens. Bioelectron. 2018, 113, 25–31. 10.1016/j.bios.2018.04.055. 29723772

[ref15] Hasan K.; Cevik E.; Sperling E.; Packer M. A.; Leech D.; Gorton L. Photoelectrochemical Wiring of Paulschulzia pseudovolvox (Algae) to Osmium Polymer Modified Electrodes for Harnessing Solar Energy. Adv. Energy Mater. 2015, 5, 1501100 10.1002/aenm.201501100.

[ref16] Yildiz H. B.; Cevik E.; Carbas B. B. Nanotechnology for Biological Photovoltaics. In Industrial Applications of Nanomaterials; Thomas S.; Grohens Y.; Pattathara Y. B., Eds.; Elsevier Inc., 2019; pp 65–89.

[ref17] Nishio K.; Hashimoto K.; Watanabe K. Light/electricity conversion by defined cocultures of Chlamydomonas and Geobacter. J. Biosci. Bioeng. 2013, 115, 412–417. 10.1016/j.jbiosc.2012.10.015. 23211438

[ref18] Tanvir R. U.; Zhang J.; Canter T.; Chen D.; Lu J.; Hu Z. Harnessing solar energy using phototrophic microorganisms: A sustainable pathway to bioenergy, biomaterials, and environmental solutions. Renewable Sustainable Energy Rev. 2021, 146, 111181 10.1016/j.rser.2021.111181. PMC8437043 34526853

[ref19] Bombelli P.; Bradley R. W.; Scott A. M.; Philips A. J.; McCormick A. J.; Cruz S. M.; Anderson A.; Yunus K.; Bendall D. S.; Cameron P. J.; Davies J. M.; Smith A. G.; Howe C. J.; Fisher A. C. Quantitative analysis of the factors limiting solar power transduction by Synechocystis sp. PCC 6803 in biological photovoltaic devices. Energy Environ. Sci. 2011, 4, 4690–4698. 10.1039/c1ee02531g.

[ref20] Gorby Y. A.; Yanina S.; McLean J. S.; Rosso K. M.; Moyles D.; Dohnalkova A.; Beveridge T. J.; Chang I. S.; Kim B. H.; Kim K. S.; Culley D. E.; Reed S. B.; Romine M. F.; Saffarini D. A.; Hill E. A.; Shi L.; Elias D. A.; Kennedy D. W.; Pinchuk G.; Watanabe K.; Ishii S.; Logan B.; Nealson K. H.; Fredrickson J. K. Electrically conductive bacterial nanowires produced by Shewanella oneidensis strain MR-1 and other microorganisms. Proc. Natl. Acad. Sci. U.S.A. 2006, 103 (30), 11358–11363. 10.1073/pnas.0604517103. 16849424 PMC1544091

[ref21] Bullen R. A.; Arnot T. C.; Lakeman J. B.; Walsh F. C. Biofuel cells and their development. Biosens. Bioelectron. 2006, 21, 2015–2045. 10.1016/j.bios.2006.01.030. 16569499

[ref22] Cevik E.; Buyukharman M.; Yildiz H. B. Construction of efficient bioelectrochemical devices: Improved electricity production from cyanobacteria (Leptolyngbia sp.) based on π-conjugated conducting polymer/gold nanoparticle composite interfaces. Biotechnol. Bioeng. 2019, 116, 757–768. 10.1002/bit.26885. 30516822

[ref23] Rosenbaum M.; He Z.; Angenent L. T. Light energy to bioelectricity: photosynthetic microbial fuel cells. Curr. Opin. Biotechnol. 2010, 21, 259–264. 10.1016/j.copbio.2010.03.010. 20378333

[ref24] Sönmezoğlu Ö. A.; Akin S.; Terzi B.; Mutlu S.; Sonmezoglu S. An Effective approach for high-efficiency photoelectrochemical solar cells by using bifunctional DNA molecules modified photoanode. Adv. Funct. Mater. 2016, 26, 8776–8783. 10.1002/adfm.201603454.

[ref25] Yildiz H. B.; Bezgin Carbas B.; Sonmezoglu S.; Karaman M.; Toppare L. A photoelectrochemical device for water splitting using oligoaniline-crosslinked [Ru(bpy)_2_(bpyCONHArNH_2_)]^+2^ dye/IrO_2_ nanoparticle array on TiO_2_ photonic crystal modified electrode. Int. J. Hydrogen Energy 2016, 41, 14615–14629. 10.1016/j.ijhydene.2016.04.249.

[ref26] Sönmezoğlu S.; Sonmezoglu O. A.; Cankaya G.; Yildirim A.; Serin N. Electrical characteristics of DNA based metal-insulator-semiconductor structures. J. Appl. Phys. 2010, 107, 124518 10.1063/1.3447985.

[ref27] Akın S.; Gulen M.; Sayin S.; Azak H.; Yildiz H. B.; Sonmezoglu S. Modification of photoelectrode with thiol-functionalized Calix[4]arenes as interface energy barrier for high efficiency in dye-sensitized solar cells. J. Power Sources 2016, 307, 796–805. 10.1016/j.jpowsour.2016.01.015.

[ref28] Ergun E. G. C.; Bezgin Carbas B. Electrochromic properties of of 2,5-dithienyl-N-substituted-pyrrole (SNS) derivatives with EDOT: Properties and electrochromic device applications. Mater. Today Commun. 2022, 32, 103888 10.1016/j.mtcomm.2022.103888.

[ref29] Aynalem B.; Muleta D. Microbial Biosensors as Pesticide Detector: An Overview. J. Sens. 2021, 2021, 5538857 10.1155/2021/5538857.

[ref30] Héquet V.; Gonzalez C.; Le Cloirec P. Photochemical processes for atrazine degradation: methodological approach. Water Res. 2001, 35 (18), 4253–4260. 10.1016/s0043-1354(01)00166-x. 11763025

[ref31] Qu M.; Liu G.; Zhao J.; Li H.; Liu W.; Yan Y.; Feng X.; Zhu D. Fate of atrazine and its relationship with environmental factors in distinctly different lake sediments associated with hydrophytes. Environ. Pollut. 2020, 256, 113371 10.1016/j.envpol.2019.113371. 31672348

[ref32] Singh S.; Kumar V.; Chauhan A.; Datta S.; Wani A. B.; Singh N.; Singh J. Toxicity, degradation and analysis of the herbicide atrazine. Environ. Chem. Lett. 2018, 16 (1), 211–237. 10.1007/s10311-017-0665-8.

[ref33] Villeneuve A.; Larroude S.; Humbert J. F. Herbicide Contamination of Freshwater Ecosystems: Impact on Microbial Communities. In Pesticides – Formulations, Effects, Fate; Stoytcheva M., Ed.; InTechOpen, 2011; pp 285–312.

[ref34] Murray K. E.; Thomas S. M.; Bodour A. A. Prioritizing research for trace pollutants and emerging contaminants in the freshwater environment. Environ. Pollut. 2010, 158, 3462–3471. 10.1016/j.envpol.2010.08.009. 20828905

[ref35] Picó Y.; Blasco C.; Font G. Environmental and food applications of LC–tandem mass spectrometry in pesticide-residue analysis: An overview. Mass Spectrom. Rev. 2004, 23, 45–85. 10.1002/mas.10071. 14625892

[ref36] Er E. Ö.; Caglak A.; Engin G. O.; Bakirdere S. Ultrasound-assisted dispersive solid phase extraction based on Fe3O4/reduced graphene oxide nanocomposites for the determination of 4-Tert octylphenol and atrazine by gas chromatography-mass spectrometry. Microchem. J. 2019, 146, 423–428. 10.1016/j.microc.2019.01.040.

[ref37] Campanale C.; Massarelli C.; Losacco D.; Bisaccia D.; Triozzi M.; Uricchio V. F. The monitoring of pesticides in water matrices and the analytical criticalities: A review. TrAC, Trends Anal. Chem. 2021, 144, 116423 10.1016/j.trac.2021.116423.

[ref38] Behniwal P. K.; She J. W. Development of HPLC-MS/MS method for the simultaneous determination of metabolites of organophosphate pesticides, synthetic pyrethroids, herbicides and DEET in human urine. Int. J. Environ. Anal. Chem. 2017, 97, 548–562. 10.1080/03067319.2017.1325881.

[ref39] Shin K. S.; Kim Y. H.; Min J. A.; Kwak S. M.; Kim S. K.; Yang E. G.; Park J. H.; Ju B. K.; Kim T. S.; Kang J. Y. Miniaturized fluorescence detection chip for capillary electrophoresis immunoassay of agricultural herbicide atrazine. Anal. Chim. Acta 2006, 573–574, 164–171. 10.1016/j.aca.2006.05.099. 17723520

[ref40] Rigo A. A.; Cezaro A. M.; Muenchen D. K.; Martinazzo J.; Manzoli A.; Steffens J.; Steffens C. Heavy metals detection in river water with cantilever nanobiosensor. J. Environ. Sci. Health B 2019, 55, 239–249. 10.1080/03601234.2019.1685318. 31680618

[ref41] Steffens C.; Ballen S. C.; Scapin E.; Maroso da Silva D.; Stefens J.; Jacques R. A. Advances of nanobiosensors and its application in atrazine detection in water: A review. Sens. Actuators Rep. 2022, 4, 100096 10.1016/j.snr.2022.100096.

[ref42] Ma Z.; Meliana C.; Munawarah H. S. H.; Karaman C.; Karimi-Maleh H.; Low S. S.; Show P. L. Recent advances in the analytical strategies of microbial biosensor for detection of pollutants. Chemosphere 2022, 306, 135515 10.1016/j.chemosphere.2022.135515. 35772520

[ref43] Lei Y.; Mulchandani P.; Chen W.; Mulchandani A. Biosensor for direct determination of fenitrothion and EPN using recombinant Pseudomonas putida JS444 with surfaceexpressed organophosphorous hydrolase. 2. Modified carbon paste electrode. Appl. Biochem. Biotechnol. 2007, 136 (3), 243–250. 10.1007/s12010-007-9023-9. 17625231

[ref44] Gäberlein S.; Spener F.; Zaborosch C. Microbial and cytoplasmic membrane-based potentiometric biosensors for direct determination of organophosphorus insecticides. Appl. Microbiol. Biotechnol. 2000, 54 (5), 652–658. 10.1007/s002530000437. 11131390

[ref45] Kumar J.; Jha S. K.; D’Souza S. F. Optical microbial biosensor for detection of methyl parathion pesticide using Flavobacterium sp. whole cells adsorbed on glass fiber filters as disposable biocomponent. Biosens. Bioelectron. 2006, 21, 2100–2105. 10.1016/j.bios.2005.10.012. 16298521

[ref46] Tucci M.; Grattieri M.; Schievano A.; Cristiani P.; Minteer S. D. Microbial amperometric biosensor for online herbicide detection: Photocurrent inhibition of Anabaena variabilis. Electrochim. Acta 2019, 302, 102–108. 10.1016/j.electacta.2019.02.007.

[ref47] Attaallah R.; Antonacci A.; Mazzaracchio V.; Moscone D.; Palleschi G.; Arduini F.; Amine A.; Scognamiglio V. Carbon black nanoparticles to sense algae oxygen evolution for herbicides detection: Atrazine as a case study. Biosens. Bioelectron. 2020, 159, 112203 10.1016/j.bios.2020.112203. 32364935

[ref48] Yildiz E.; Camurlu P.; Tanyeli C.; Akhmedov I.; L Toppare L. A soluble conducting polymer of 4-(2,5-di(thiophen-2-yl)-1H-pyrrol-1-yl)benzenamine and its multichromic copolymer with EDOT. J. Electroanal. Chem. 2008, 612, 247–256. 10.1016/j.jelechem.2007.10.004.

[ref49] Dervisevic M.; Dervisevic E.; Senel M.; Cevik E.; Yildiz H. B.; Camurlu P. Construction of ferrocene modified conducting polymer based amperometric urea biosensor. Enzyme Microb. Technol. 2017, 102, 53–59. 10.1016/j.enzmictec.2017.04.002. 28465061

[ref50] Yildiz H. B.; Tel-Vered R.; Willner I. Solar Cells with Enhanced Photocurrent Efficiencies Using Oligoaniline-Crosslinked Au/CdS Nanoparticles Arrays on Electrodes. Adv. Funct. Mater. 2008, 18, 3497–3505. 10.1002/adfm.200800810.

[ref51] Carbas B. B.; Guler M.; Yucel K.; Yildiz H. B. Construction of novel cyanobacteria-based biological photovoltaic solar cells: Hydrogen and photocurrent generated via both photosynthesis and respiratory system. J. Photochem. Photobiol. A 2023, 442, 114764 10.1016/j.jphotochem.2023.114764.

[ref52] Christenson A.; Shleev S.; Mano N.; Heller A.; Gorton L. Redox potentials of the blue copper sites of bilirubin oxidases. Biochim. Biophys. Acta 2006, 1757 (12), 1634–1641. 10.1016/j.bbabio.2006.08.008. 17020746

[ref53] Hasan K.; Yildiz H. B.; Sperling E.; O′Conghaile P.; Packer M. A.; Leech D.; Hagerhall C.; Gorton L. Photo-electrochemical communication between cyanobacteria (Leptolyngbia sp.) and osmium redox polymer modified electrodes. Phys. Chem. Chem. Phys. 2014, 16, 24676–24680. 10.1039/C4CP04307C. 25325401

[ref54] Yan Y. M.; Baravik I.; Tel-Vered R.; Willner I. An ethanol/O_2_ biofuel cell based on an electropolymerized bilirubin oxidase/Pt nanoparticle bioelectrocatalytic O_2_-reduction cathode. Adv. Mater. 2009, 21, 4275–7279. 10.1002/adma.200900206.

[ref55] Çevik E.; Titiz M.; Senel M. Light-dependent photocurrent generation: Novel electrochemical communication between biofilm and electrode by ferrocene cored poly(amidoamine) dendrimers. Electrochim. Acta 2018, 291, 41–48. 10.1016/j.electacta.2018.08.108.

[ref56] Scognamiglio V.; Raffi D.; Lambreva M.; Rea G.; Tibuzzi A.; Pezotti G.; Johanningmeier U.; Giardi M. T. Chlamydomonas reinhardtii genetic variants as probes for fluorescence sensing system in detection of pollutants. Anal. Bioanal. Chem. 2009, 394, 1081–1087. 10.1007/s00216-009-2668-1. 19238365

[ref57] Shitanda I.; Takamatsu S.; Watanabe K.; Itagaki M. Amperometric screen-printed algal biosensor with flow injection analysis. Electrochim. Acta 2009, 54, 4933–4936. 10.1016/j.electacta.2009.04.005.

