# Supplementary material for: Construction of a Conductive Polymer/AuNP/Cyanobacteria-Based Biophotovoltaic Cell Harnessing Solar Energy to Generate Electricity via Photosynthesis and Its Usage as a Photoelectrochemical Pesticide Biosensor: Atrazine as a Case Study
Source: ACS Omega. 2024 Mar 27;9(14):16249–61. doi: 10.1021/acsomega.3c10308 (PMC11007689; doi:10.1021/acsomega.3c10308)
Supplement: Supplementary file 1 — ao3c10308_si_001.pdf [file ao3c10308_si_001.pdf]

Construction of a Conductive Polymer/AuNP/Cyanobacteria-Based Biophotovoltaic Cell Harnessing Solar Energy To Generate Electricity via Photosynthesis and Its Usage as a Photoelectrochemical Pesticide Biosensor: Atrazine as a Case Study

Mustafa Buyukharman[1], Ibrahim Ender Mulazimoglu[2], Huseyin Bekir Yildiz*[3,4]

[1] Department of Physics, Faculty of Science, Istanbul University, TR-34134 Istanbul, Turkey

[2] Department of Chemistry, Ahmet Kelesoglu Education Faculty, Necmettin Erbakan University, TR-42090 Konya, Turkey

[3] Department of Mechanical Engineering, Faculty of Engineering Architecture and Design, Bartin University, TR-74100 Bartin, Turkey

[4] Photo-Electrochemical Systems and Materials Research Group, The Central Research Laboratory-Research and Application Center, Bartin University, TR-74100 Bartin, Turkey

Keywords: Biophotovoltaics, Conductive polymers, Cyanobacteria, Atrazine, Biosensor

Synthesis and characterization of 4-(2,5-di(thiophen-2-yl)-1H-pyrrol-1-yl)benzamine (SNS-Aniline) monomer

As stated in the literature [2], 1,4-di(2-thienyl)-1,4-butanedione, one of the reactants of SNS-Aniline monomer, is the result of the Friedel-Crafts reaction of thiophene and succinyl chloride catalyzed by aluminum chloride ($AlCl_3$) obtained with its use. Then, 1,4-di(2-thienyl)-1,4-butanedione and benzene-1,4-diamine obtained from this reaction are reacted with each other in toluene solution catalyzed by propionic acid and 4-(2,5-di(thiophene-2) -yl)-1-H-pyrrol-1-yl) benzenamine (SNS-Ph-NH_2) monomer was synthesized with a 41 % yield. (**Scheme SI-1**). ^{1}H-NMR ($CDCl_3$) (δ/ppm): 3.75 (s, 2H), 6.46 (dd, 2H), 6.58 (s, 2H), 6.73 (dd, 2H), 6.80 (d, 2H), 6.97 (dd, 2H), 7.12 (d, 2H). ^{13}C-NMR ($CDCl_3$) (δ/ppm): 109.5, 115.1, 124.2, 124.4, 124.5, 127.0, 129.1, 130.3, 135.7, and 146.5.

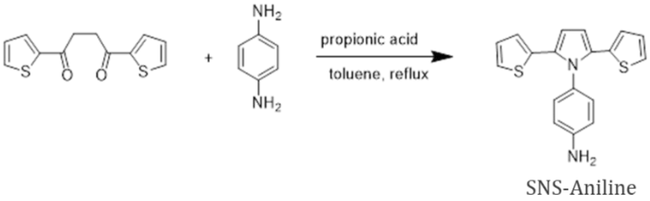

Scheme S1. Synthesis route for SNS-Aniline monomer

Homopolymerization of SNS-Aniline monomer

Homopolymerization of SNS-NH_2 took place in a single chamber cell containing 3.10^{-2} M SNS-NH_2 in the medium of 0.1 M $NaClO_4$/0.1 M $LiClO_4$/acetonitrile solution. After potentiodynamic electrochemical polymerization, P(SNS-Ph-NH_2), $NaClO_4$/$LiClO_4$ was washed with acetonitrile to remove unreacted monomers. Gold electrode as working electrode, platinum wire as counter electrode and Ag/AgCl electrode alternating voltammetry measurements as reference electrode were used. With the reversible voltammetry studies, an irreversible oxidation peaks were detected at 0.55 V and 0.66 V of the monomer in 0.1 M $NaClO_4$/0.1 M $LiClO_4$/acetonitrile solution. This indicates that the monomer radical cation, which is necessary for the synthesis of conductive polymers by electrochemical method, can be formed. In addition, as the number of cycles increased, the presence of new redox peaks belonging to the conductive polymer coated on the electrode was detected [1].

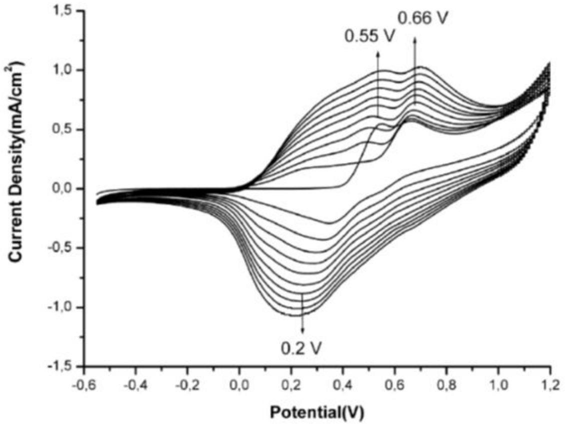

Figure S1. Cyclic voltammogram of its monomer in 0.1 M $NaClO_4$/0.1 M $LiClO_4$/acetonitrile solution with scan rate of 100 mV/s in N_2 atmosphere (Working and Counter electrode: Pt wire, Reference electrode: Ag/AgCl electrode).

Synthesis of Thioaniline Functionalized Gold Nanoparticles

Au NPs functionalized with 2-mercaptoethane sulfonic acid and p-aminothiophenol were added with 10 mL of ethanol solution containing 197 mg of $HAuCl_4$ and 5 mL of methanol solution containing 42 mg of mercaptoethane sulfonate and 8 mg of p-aminothiophenol into 2.5 mL of glacial acetic acid, and this new solution was dissolved in 2.5 mL of glacial acetic acid. It was prepared by mixing in an ice bath for 1 h [49]. Then, 1 M 7.5 mL of $NaBH_4$ solution was added dropwise to this solution until a black solution was obtained. The new black solution was

stirred in the ice bath for another 1 h, then removed from the ice bath and stirred at room temperature for another 14 h. The resulting nanoparticles were washed twice with methanol, ethanol and diethyl ether solutions and then centrifuged. These Au NPs were characterized by transmission electron microscopy. When examined by scanning transmission electron microscopy (STEM) and field emission scanning electron microscope(FE-SEM), the dimensions of the particles were estimated to be 8.5 nm (Figure SI- 2) [2].

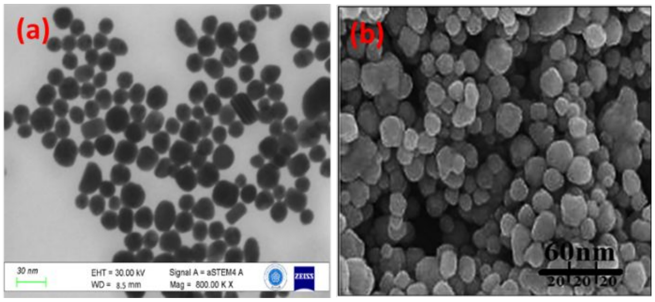

Figure S2. a) Scanning Transmission Electron Microscope (STEM) (Scale 30 nm) and b) Field Emission Scanning Electron Microscope (FESEM) (Scale 60 nm) images of thioaniline functionalized AuNPs.

The presence of aniline group belonging to AuNPs on the P(SNS-Aniline) mofidifed gold electrode can be explained as electrochemically. Thioaniline functionalized AuNPs were electrochemically polymerized using the electropolymerization method in a phosphate buffer solution at pH 7.4. The polymerization occurred within a potential range of -0.5 V to +0.5 V using a cyclic voltammetry technique. The resulting polymer was attached to the previously electropolymerized SNS-Aniline polymer on a gold electrode surface using the same electropolymerization method. Figure S3 illustrates the cyclic voltammogram obtained after the electrochemical attachment of AuNPs to P(SNS-Aniline). In the cyclic voltammogram, two quasi-reversible redox waves were observed. The first redox wave, centered at 0.1 V using a Ag/AgCl as the reference electrode (Eox 0.145 V vs Ag/AgCl and Ered 0.04 V vs AgCl), was attributed to the redox reaction of bisaniline cross-linking units. The second redox wave centered at -0.30 V (Eox -0.20 V vs AgCl; Ered -0.36 V vs AgCl) was explained as the binding of Ag+ ions to the P(SNS-Aniline) modified surface (Figure S3). The graph in the figure depicts the peak potentials corresponding to the oxidation of aniline dimer cross-linking units at different pH points [3, 4].

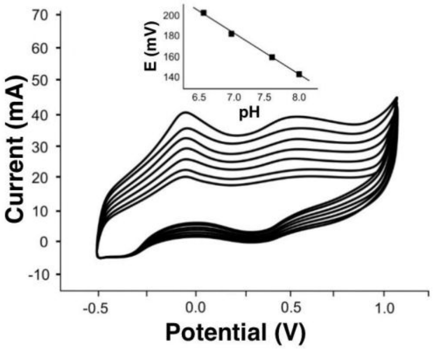

Figure S3. The cyclic voltammogram of thioaniline functionalized AuNPs attached to P(SNS-NH2) through electrochemical polymerization. This voltammogram was obtained in an electrolyte solution containing 0.1 M phosphate buffer (pH=7.4) under argon gas, and measurements were taken at a scan rate of 100 mV/s.

REFERENCES

[1] Yildiz, E., Camurlu, P., Tanyeli, C., Akhmedov, I., Toppare, L. A soluble conducting polymer of 4-(2,5-di(thiophen-2-yl)-1H-pyrrol-1-yl)benzenamine and its multichromic copolymer with EDOT. J. Electroanal. Chem. **2008**, 612, 247–256.

[2] Yildiz, H.B., Tel-Vered, R., Willner, I. Solar Cells with Enhanced Photocurrent Efficiencies Using Oligoaniline-Crosslinked Au/CdS Nanoparticles Arrays on Electrodes. Adv. Funct. Mater. **2008**, 18, 3497–3505

[3] Granot E., Patolsky F., Willner I. Electrochemical Assembly of a CdS Semiconductor Nanoparticle Monolayer on Surfaces: Structural Properties and Photoelectrochemical Applications, J. Phys. Chem. B 2004, 108, 5875-5881.

[4] Tel-Vered, R., Yildiz H.B., Willner I.. Enhanced Photocurrents Generated by Supramolecular Relay/CdS-Nanoparticle/Electron-Donor Structures on Gold Electrodes, Adv. Mater. 2009, 21,716-720.
